# High-quality chronic care delivery improves experiences of chronically ill patients receiving care

**DOI:** 10.1093/intqhc/mzt065

**Published:** 2013-10-11

**Authors:** Jane Murray Cramm, Anna Petra Nieboer

**Affiliations:** Institute of Health Policy & Management (iBMG), Erasmus University, Rotterdam, The Netherlands

**Keywords:** chronic care, disease management, quality, integrated care

## Abstract

**Objective:**

Investigate whether high-quality chronic care delivery improved the experiences of patients.

**Design:**

This study had a longitudinal design.

**Setting and Participants:**

We surveyed professionals and patients in 17 disease management programs targeting patients with cardiovascular diseases, chronic obstructive pulmonary disease, heart failure, stroke, comorbidity and eating disorders.

**Main Outcome Measures:**

Patients completed questionnaires including the Patient Assessment of Chronic Illness Care (PACIC) [T1 (2010), 2637/4576 (58%); T2 (2011), 2314/4330 (53%)]. Professionals' Assessment of Chronic Illness Care (ACIC) scores [T1, 150/274 (55%); T2, 225/325 (68%)] were used as a context variable for care delivery. We used two-tailed, paired *t*-tests to investigate improvements in chronic illness care quality and patients' experiences with chronic care delivery. We employed multilevel analyses to investigate the predictive role of chronic care delivery quality in improving patients' experiences with care delivery.

**Results:**

Overall, care quality and patients' experiences with chronic illness care delivery significantly improved. PACIC scores improved significantly from 2.89 at T1 to 2.96 at T2 and ACIC-S scores improved significantly from 6.83 at T1 to 7.18 at T2. After adjusting for patients' experiences with care delivery at T1, age, educational level, marital status, gender and mental and physical quality of life, analyses showed that the quality of chronic care delivery at T1 (*P* < 0.001) and changes in care delivery quality (*P* < 0.001) predicted patients' experiences with chronic care delivery at T2.

**Conclusion:**

This research showed that care quality and changes therein predict more positive experiences of patients with various chronic conditions over time.

## Introduction

Due to an aging population and greater longevity, the prevalence of chronic conditions is rising [1]. This growth has placed increasing demands on health-care systems and resulted in deficiencies in the organization and delivery of chronic care [2–4]. Care delivery is often poorly organized and health-care organizations face constraints in the use of modern information technology [5]. Although many advances have been made in the treatment available to chronically ill patients, these patients do not always receive optimal care [5–12]. Health-care delivery often focuses on acute problems and rapid short-term solutions, without the initiation of chronic professional treatment or the active involvement of chronically ill patients [13]. Historically, health-care delivery did not focus on enhancing patients' self-management abilities because the full clinical course of acute diseases often encompasses a period of days or a few weeks [1]. Today, care delivery to chronically ill patients remains acute driven in many health-care practices, and system design has been identified as a fundamental barrier to quality improvement [9–12].

The delivery of effective and high-quality chronic care requires comprehensive system changes that entail more than simply implementing interventions or adding new features to the existing acute-focused system [10–12]. Wagner *et al*. [1] developed the chronic care model to guide quality improvement in chronic care delivery by providing examples of how health-care practices can shift fundamentally from acute and reactive care to care that is organized, structured, planned, patient centered and proactive through a combination of effective multidisciplinary team care and planned interactions with patients. Important elements of the model include strengthening the provider–patient relationship and improving health outcomes through self-management support, effective use of community resources, integrated decision support for professionals, and the use of patient registries and other supportive information technology [1, 14]. A recent literature review reaffirmed the notion that successful improvement strategies in chronic disease care are consistent with the concept of the chronic care model [14].

Bonomi *et al*. [15] developed the Assessment of Chronic Illness Care (ACIC) to assess professionals' perceptions of the quality of chronic care delivery. The ACIC is based on six areas of systemic change suggested by the chronic care model and was developed to help organizational teams identify areas requiring improvement and to evaluate the level and nature of improvements made in their system [15, 16]. In addition, Glasgow *et al*. [17] developed the Patient Assessment of Chronic Illness Care (PACIC) to assess patients' experiences with chronic care delivery. The PACIC has been used internationally among patients with a variety of chronic health conditions, including diabetes, osteoarthritis, depression, asthma, hypertension and chronic obstructive pulmonary disease (COPD) [18–22]. Although available studies have reported on the quality of chronic care, as assessed by the ACIC [15, 16, 23–35], or on experiences with care delivery, as assessed by the PACIC [18–22], it remains unclear whether high-quality chronic care delivery improves the experiences of chronically ill patients receiving such care. Furthermore, many studies have included only patients with a single chronic disease and most have used a cross-sectional design.

Therefore, this research aimed to investigate whether high-quality chronic care delivery improved the experiences of patients with various chronic conditions over time by jointly assessing professionals' perceptions (ACIC scores) and patients' experiences (PACIC scores). We focused on disease management programs based on the chronic care model, which may serve as examples of systemic change that improves the efficiency and effectiveness of chronic care delivery [8] by combining patient-related, professionally directed and organizational interventions [26, 27]. Because changes in the system of chronic care delivery are experienced first by professionals, followed by patients, we expected that higher quality chronic care delivery, as perceived by professionals, would be associated with improved experiences of their chronically ill patients.

## Methods

A national program on disease management for chronic illnesses carried out by the Netherlands Organization for Health Research and Development and commissioned by the Dutch Ministry of Health provided funding for practices planning a redesign of primary care according to the chronic care model. To qualify for funding, practices also had to have both experience in chronic care delivery and the ability to implement all systems required for such delivery. Twenty-two out of a possible 38 practices met all criteria.

In 2010, most disease management programs had finished implementing interventions (e.g. information and communication technologies systems, training of professionals, care protocols, redistribution of tasks) and had started to enroll patients. All patients included in the disease management programs and professionals involved were surveyed during the beginning of the program in 2010 and 1 year later in 2011.

This study was approved by the ethics committee of the Erasmus University Medical Centre, Rotterdam (September 2009).

### Participants

This study included professionals and patients in 17 disease management programs (out of 22 disease management programs) based on the chronic care model that were implemented in various Dutch regions. At the time of the survey these disease management programs did not receive structural financial support for their disease management programs yet (e.g. through bundled payments). Reasons for excluding five disease management programs were as follows: (i) small sample size in one project (<15 patients); (ii) different timing of distribution of questionnaire in one project (just finished T1 and T2 data are not available yet) and (iii) three projects already delivered chain care at T1, before implementing the disease management program. These programs implemented disease management interventions in 110 healthcare practices and 2 hospitals in 17 regions in the Netherlands and were characterized by a variety of collaborations, such as those between general practitioners (GPs) and hospitals and among primary care team members (e.g. pharmacists, physiotherapists, dieticians, social workers). The 17 projects targeted populations of patients with cardiovascular diseases (*n* = 9), COPD (*n* = 4), heart failure (*n* = 1), stroke (*n* = 1), comorbidity (aimed at patients with cardiovascular diseases and diabetes or patients with COPD and heart failure) (*n* = 1) and eating disorders (*n* = 1) [28–30].

### Measures

We used the ACIC Short version (ACIC-S) to investigate professionals' assessment of chronic care delivery [16]. The ACIC-S consists of 21 items covering the 6 areas of the chronic care model: healthcare organization (*n* = 3), community linkages (*n* = 3), self-management support (*n* = 3), delivery system design (*n* = 3), decision support (*n* = 3) and clinical information systems (*n* = 3). Additional items integrate the six components, such as by linking patients' self-management goals to information systems (*n* = 3). Responses to ACIC Short version items (e.g. ‘evidence-based guidelines are available and supported by provider education’) fall within four descriptive levels of implementation ranging from ‘little or none’ to ‘fully implemented intervention’. Within each of the four levels, respondents are asked to choose the degree to which that description applied. The result is a 0–11 scale, with categories defined as 0–2 (little or no support for chronic illness care), 3–5 (basic or intermediate support), 6–8 (advanced support) and 9–11 (optimal or comprehensive integrated care for chronic illness). Subscale scores for the areas of the chronic care model are derived by calculating the average score for all items in that subsection of items. Mean subscale scores were calculated if at least two out of three items were available. Total scale scores were calculated by average scores on the subsections (when at least four out of seven subsections were available). Cronbach's alpha of the ACIC was 0.90 at T0 and 0.89 at T1, indicating reliability. Mean scores of all professionals at each of the 17 disease management programs were calculated and aggregated to the disease management program level as a context variable for all patients enrolled in the 17 disease management programs.

We used the PACIC to assess patients' perspectives on the quality of chronic care delivery. The PACIC consists of 20 items, with a five-point response scale ranging from 1 (almost never) to 5 (almost always). The PACIC score is obtained by dividing the sum of each participant's responses by 20. Scores thus ranged from 1 to 5, with higher scores indicating a better perception of chronic care delivery. Cronbach's alpha of the overall PACIC scale was 0.93 at T1 and T2 (2011).

Patients' physical and mental quality of life were assessed with the Short Form 36 Health Survey (SF-36). Rules for item scoring and scales are available in the SF-36 scoring manual [31]. All scales were transformed to values between 0 and 100 to allow comparison among patient groups. Physical and mental component scores were calculated. Selected items and weights derived from the general Dutch population were then used to score the physical and mental quality of life components [32], with higher scores indicating more positive ratings.

Patients' educational levels were assessed on six levels ranging from 1 [no school or primary education (≤7 years)] to 6 [university degree (≥18 years)]. We dichotomized this item into low (no school or primary education) or high (more than primary education) educational level.

### Survey administration

At T1 (2010), the ACIC-S survey instrument was distributed to 274 professionals participating in the 17 disease management programs and completed by 150 respondents (55% response rate). One year later in 2011 (T2), the ACIC-S was distributed to 325 professionals participating in the 17 disease management programs and completed by 225 respondents (68% response rate). A total of 102 respondents filled in the ACIC-S questionnaire at both T1 and T2. At T1 and T2, questionnaires were distributed to potential respondents through a contact person at each participating organization (through internal mailboxes or personal delivery at team meetings) or by direct mailing. Two weeks later, the same procedure was used to send a reminder to non-respondents.

At T1 (2010), questionnaires were distributed to all 4576 patients participating in the 17 disease management programs and completed by 2637 respondents (58% response rate). One year later in 2011 (T2), questionnaires were distributed to all 4330 patients still participating in the 17 disease management programs and completed by 2314 respondents (53% response rate). A total of 1293 respondents filled in the questionnaire at both T1 and T2. At T1, most questionnaires were mailed to patients' homes and some were distributed by professionals working in these practices during consultations. However, not all patients were seen by professionals within a short period of time, prompting us to change our strategy and mail questionnaires to all patients' homes at T2. A few weeks after initial distribution, a reminder notice and another copy of the questionnaire were sent to non-respondents. No incentive in the form of money or gifts was offered to professionals or patients for participation in the survey.

### Statistical analyses

We used descriptive statistics to describe the study population. ACIC-S scores served as a organizational level characteristic for care delivery to patients. Two-tailed, paired *t*-tests were used to investigate improvements in the quality of chronic illness care and patients' experiences with chronic care delivery over time. Quality of care and patients' experiences with care delivery were compared among the 17 disease management programs using analysis of variance. Preliminary testing revealed that the disease management program level affected patients' experiences with chronic care delivery (−2 log likelihood, 4812.204 Model 0 vs. 4765.117 Model 1; *P* = 0.01). Therefore, to account for the hierarchical structure of the study design, we fitted a hierarchical random-effects model of 1000 patients (level 1) nested in 17 disease management programs (level 2) using a random-intercept effect. We employed this multilevel model to investigate the predictive role of chronic care delivery quality in patients' improved experiences with care delivery. We first estimated an empty model (0) that reflected variation in the intercept. To assess the extent to which variance should be ascribed to the higher disease management program level rather than to the individual level, disease management programs were then introduced as level-2 units (Model 1). In the final full model (2), we introduced the explanatory variables (quality of chronic care delivery at T1 and changes in quality of chronic care delivery) while controlling for patients' experiences with chronic care delivery at T1, age, educational level, marital status and mental and physical quality of life. The −2 log likelihood of Model 2 (2088.004) was significantly better compared with Model 1 (4765.117; *P* = 0.01). In addition, we tested the final full model on imputated data [10 imputated data sets using the Monte Carlo Marcov chain (MCMC) method]. Results were considered statistically significant if two-sided *P* values were ≤0.05.

## Results

Table 1 displays characteristics of the patient sample at T1. Of the 2637 respondents, 49% were female, 41% had a low educational level and 29% were single. Mean age was 65.03 ± 12.10 (range, 18–92) years. The mean physical quality of life was 42.10 ± 10.38 and mental quality of life was 48.60 ± 10.48. We tested for differences between respondents who completed only one questionnaire and those who completed both questionnaires. No differences were found in patients' experiences with chronic care delivery, marital status, physical quality of life and gender. On average, respondents who completed T0 only were younger (64.15 ± 11.07 vs. 65.43 ± 9.94 years; *P* < 0.01), reported lower mental quality of life (48.27 ± 10.33 vs. 49.78 ± 9.59; *P* < 0.001) and were lower educated compared with those who completed both questionnaires (44 vs. 40%; *P* < 0.05).

**Table 1:** Characteristics of patients participating in disease management programs at T1

	Patients (*n* = 2637)
Mean age (years)	65.03 ± 12.10 (18–92)
Gender (female)	49%
Marital status (single)	29%
Educational level (low)	41%
Physical quality of life (SF-36)	42.10 ± 10.38 (11–70)
Mental quality of life (SF-36)	48.60 ± 10.48 (3–73)

SF-36, Short Form 36 Health Survey. Data are expressed as mean ± standard deviation (range) or percentage.

The majority of professionals at T1 (61%) was female and the mean age was 46.4 ± 9.8 years (range: 23–64 years). Respondents consisted primarily of GPs (39%), practice nurses (30%), policy and management personnel (9%) and paramedical staff (9%).

The mean quality of chronic care delivery (ACIC-S) score at T1 for all disease management programs was 6.83, indicating reasonably good support for chronic illness care. Paired *t*-test results showed significant improvement in all quality of chronic care delivery areas as assessed with the ACIC-S at T2. The mean ACIC-S score at T2 was 7.18, still indicating reasonably good support for chronic illness care. The same picture emerged for patients' experiences with chronic care delivery; PACIC scores improved significantly from 2.89 at T1 to 2.96 at T2 (Table 2). Practice design was the only PACIC subscale that did not improve over time.

**Table 2:** Changes in quality of chronic care delivery and patients' experiences with chronic care delivery over time (*n* = 1143)

	Baseline (T1) assessment		Follow-up (T2) assessment		T2−T1		P*
	M	SD	M	SD	M	SD	
Quality of chronic care delivery							
Organization of the health-care delivery system	7.44	(1.06)	7.53	(0.79)	0.09	(0.98)	<0.001
Community linkages	6.73	(0.85)	6.89	(0.67)	0.16	(0.66)	<0.001
Self-management support	6.47	(1.37)	6.71	(1.06)	0.24	(0.92)	<0.001
Decision support	7.13	(0.93)	7.20	(0.75)	0.08	(0.58)	<0.001
Delivery system design	7.37	(0.67)	8.36	(0.48)	0.99	(0.55)	<0.001
Clinical information systems	6.51	(1.23)	6.80	(0.75)	0.29	(0.94)	<0.001
Integration of chronic care components	6.33	(1.24)	6.78	(0.82)	0.48	(1.04)	<0.001
Overall ACIC score	6.83	(0.94)	7.18	(0.64)	0.35	(0.61)	<0.001
Patients' experiences with quality of care							
Patient activation	3.01	(1.16)	3.11	(1.12)	0.10	(1.20)	<0.01
Practice design	3.56	(0.95)	3.53	(0.93)	−0.03	(0.96)	0.304
Goal setting/tailoring	2.75	(0.95)	2.83	(0.94)	0.08	(0.96)	<0.01
Problem-solving	2.85	(1.12)	2.95	(1.09)	0.10	(1.10)	<0.01
Follow-up/coordination	2.29	(0.95)	2.35	(0.97)	0.07	(1.00)	<0.05
Overall PACIC score	2.89	(0.84)	2.96	(0.86)	0.07	(0.82)	<0.01

M, mean; SD, standard deviation; ACIC-S, Assessment of Chronic Illness Care Short version; PACIC, Patient Assessment of Chronic Illness Care.

*Paired *t*-test, T1 vs. T2.

Patients' perceptions of changes in the quality of chronic care delivery, as assessed with the PACIC did not vary among disease management programs (*F*_group_ = 1.6; *P* = 0.060; Table 3). At disease management program level significant changes were found in Regionale Organisatie Huisartsen Amsterdam (CVD), Stichting Gezondheidscentra Eindhoven (CVD), Sint Lucas Andreas (Stroke) and Ursula (Eating disorders) (*P* < 0.10).

**Table 3:** Changes in Patient Assessment of Chronic Illness Care Scores (T2–T1)

Disease management program	*n*	Mean	SD
CVD: Onze Lieve Vrouwe Gasthuis	69	0.03	0.79
CVD: Stichting Eerstelijns Samenwerking Achterveld	46	0.13	0.94
CVD: Regionale Organisatie Huisartsen Amsterdam	48	0.19	0.74
CVD: Stichting Gezondheidscentra Eindhoven	52	0.20	0.73
CVD: Gezondheidscentrum Maarssenbroek	96	0.12	0.66
CVD: Rijnstate	125	0.00	0.95
CVD: Medical Centre Oud-West	14	0.25	0.89
CVD: University Medical Centre St. Radboud	71	0.13	0.95
CVD: Huizen	79	0.08	0.69
Heart failure: Hafank	19	−0.22	0.67
Stroke: Sint Lucas Andreas	28	0.58	1.06
COPD: Zorgnetwerk Midden-Brabant	87	0.00	0.69
COPD: Archiatros	166	0.02	0.79
COPD: Monnickendam	50	−0.02	0.77
COPD: Almere	55	0.09	0.81
Comorbidity: Pantein	100	−0.08	0.81
Eating disorders: Ursula	38	0.23	0.75
Total	1143	0.07	0.82

SD, standard deviation; CVD, cardiovascular disease; COPD, chronic obstructive pulmonary disease. Analysis of variance for change in Patient Assessment of Chronic Illness Care scores: *F*_group_ = 1.6; *P* = 0.060. Comorbidity disease management program is aimed at patients with cardiovascular diseases and diabetes or patient with COPD with heart failure.

The results of multilevel analyses are displayed in Table 4. After adjusting for patients' experiences with chronic care delivery at T1, age, educational level, marital status, gender and quality of life (mental and physical components), these analysis showed that the quality of chronic care delivery at T1 (*P* < 0.001) and changes in the quality of chronic care delivery (*P* < 0.001) predicted patients' experiences with chronic care delivery at T2. The intraclass correlation (ICC  =  0.05) revealed an appreciable clustering of individuals within the disease management programs, showing that 5% of the total individual differences in patients' perceptions of quality of care occurred at the disease management level and might be attributable to contextual factors or to the different design of the programs. Multilevel analyses on imputated data (10 imputated data sets using the MCMC method) showed similar results: patients' experiences with chronic care delivery at T1 (PACIC), quality of chronic care delivery at T1 (ACIC-S) and changes in the quality of chronic care delivery predicted patients' experiences with chronic care delivery at T2.

**Table 4:** Predictors of patients’ experiences with chronic care delivery, as assessed by multilevel regression analyses (*n* = 1000)

Model	0		1		2	
	*B*	SE	*B*	SE	*B*	SE
Constant	2.95	0.02	2.95	0.05	0.98	0.32
Patients’ experiences with chronic care delivery at T1 (PACIC)					0.51*	0.03
Age					−0.01*	0.00
Low educational level					0.08	0.05
Marital status (single)					−0.08	0.05
Gender (female)					−0.08	0.05
Quality of life mental component (SF-36)					0.00	0.00
Quality of life physical component (SF-36)					−0.00	0.00
Quality of chronic care delivery at T1 (ACIC-S)					0.15*	0.04
Changes in quality of chronic care delivery (ACIC-S)					0.23*	0.05
−2 log likelihood	4812.204		4765.117		2088.004	

SE, standard error; T1, baseline (2010); PACIC, Patient Assessment of Chronic Illness Care; SF-36, Short Form 36 Health Survey; ACIC-S, Assessment of Chronic Illness Care Short version. Model 0 is the null model including the dependent variable only, without the multilevel structure. Model 1 is the empty model (random effects), which includes the dependent variable with the multilevel structure, but without explanatory variables. In Model 2 the explanatory variables enter the equation.

*P ≤ 0.001 (two-tailed).

## Discussion

This study aimed to investigate whether high-quality chronic care delivery improved chronically ill patients' experiences with the delivery of such care. Overall, this research clearly showed that both the quality of care and changes in chronic care delivery predicted more positive experiences of chronically ill patients. Some differences, however, stand out. The heart failure, comorbidity and Monnickendam COPD disease management programs were not able to improve the quality of care delivery, although their decrease is not significant. Previous meta-analyses and reviews have also reported heterogeneity in the effectiveness of disease management programs for patients with COPD and heart failure that they ascribed to several factors such as differences in study quality and the length of follow-up [33–35]; however, the 17 disease management programs in our study had the same length of follow-up and were assessed using the same study design. Mackenzie *et al*. [36] additionally identified a negative relationship between the severity of chronic diseases and quality of care, which may explain the decline in the quality of care observed in three disease management programs in this study, which included patients with greater disease severity, namely patients with heart failure, comorbidity and severe COPD. The health condition of patients with heart failure is known to decline rapidly [37], and this disease management program had the largest attrition rate due to death among the Dutch disease management programs examined here (13 vs. less than 1% in the other disease management programs that was reported back to us). Providing high-quality care for such a highly burdened patient population in the primary care setting may be difficult, and more intensified care may be needed. The same argument may apply to the comorbidity disease management program; delivering high-quality care to patients with multiple chronic conditions may be difficult due to their complex needs. Lastly, one of the four COPD disease management programs examined in this study did not improve the quality of care; this program included COPD patients with Global Initiative for Obstructive Lung Disease (GOLD) stages 1–4 (classification of pulmonary function: 1 = mild, 2 = moderate, 3 = severe, 4 = very severe), whereas the other three programs included only COPD patients with GOLD stages 1 and 2. Thus, including patients with more severe COPD may explain the decline in the quality of care delivery in this disease management program. Disease management programs may find it more difficult to enhance or even maintain the quality of care for patients with more severe diseases as the diseases progress and the patients' health status deteriorates. These patients may require a case-management type of care or an intensified disease management program. Although stroke also has a major impact on patients' health, their health status in most cases improves after the initial event, which may explain the ability of this disease management program to improve the quality of care.

The study has several limitations. First, because it did not involve a control group, we were unable to determine whether improvements in the quality of care delivery were caused by the disease management programs or other factors. Secondly, because this study included patients enrolled in disease management programs based on the chronic care model, our findings apply to only similar disease management programs, and not, for example, to commercialized disease management programs. Thirdly, small numbers of patients participated in some disease management programs, and the results should thus be interpreted with caution. Fourthly, from our research findings we hypothesize that disease severity might be an explanation. Future research is necessary to verify the possible effect of disease severity. Fifthly, we investigated individual level and disease management level only, other levels (such as organizational level) may also be relevant. Sixthly, we found differences between respondents who completed T1 questionnaires only vs. those who completed both questionnaires (T1 and T2) regarding their age, mental quality of life and educational level. Since we correct for background characteristics and quality of life in the multilevel analyses this however did not affect our main study finding that both the quality of care and changes in chronic care delivery predicted more positive experiences of chronically ill patients. Finally, although we found a statistically significant difference in patients' experiences with care delivery over time, the difference found is relatively small and may not be clinically relevant.

## Conclusions

This research clearly showed that both the quality of care and changes in chronic care delivery predicted more positive experiences of chronically ill patients. These findings are especially important in a time of aging populations and increasing prevalence of people with chronic conditions. Redesigning care systems and implementing disease management programs based on the chronic care model may contribute to improved quality of care and patients' experiences with chronic care delivery.

## Funding

This work was supported by The Netherlands Organisation for Health Research and Development (ZonMw), a national organisation that promotes quality and innovation in the field of health research and health care, initiating and fostering new developments (ZonMw project number 300030201). Funding to pay the Open Access publication charges for this article was provided by the Netherlands Organization for Health Research and Development (ZonMw), a national organization that promotes quality and innovation in the field of health research and health care, initiating and fostering new developments (ZonMw project number 300030201).
